# Increased levels of VEGF-C and macrophage infiltration in lipedema patients without changes in lymphatic vascular morphology

**DOI:** 10.1038/s41598-020-67987-3

**Published:** 2020-07-02

**Authors:** Gunther Felmerer, Aikaterini Stylianaki, Maija Hollmén, Philipp Ströbel, Adam Stepniewski, Anna Wang, Florian S. Frueh, Bong-Sung Kim, Pietro Giovanoli, Nicole Lindenblatt, Epameinondas Gousopoulos

**Affiliations:** 1 Division of Plastic Surgery, Department of Trauma Surgery, Orthopaedics and Plastic Surgery, University Medical Center Göttingen, Georg-August-University, Göttingen, Germany; 2 Department for Plastic, Aesthetic and Hand Surgery, General Hospital Braunschweig, Brunswick, Germany; 3 MediCity Research Laboratory, University of Turku, Turku, Finland; 4 Institute of Pathology, University Medical Center Göttingen, Georg-August-University, Göttingen, Germany; 5 Department of Plastic Surgery and Hand Surgery, University Hospital Zurich, Rämistrasse 100, 8091 Zurich, Switzerland

**Keywords:** Translational research, Obesity, Oedema, Molecular medicine

## Abstract

Lipedema is a chronic adipose tissue disorder characterized by the disproportional subcutaneous deposition of fat and is commonly misdiagnosed as lymphedema or obesity. The molecular determinants of the lipedema remain largely unknown and only speculations exist regarding the lymphatic system involvement. The aim of the present study is to characterize the lymphatic vascular involvement in established lipedema. The histological and molecular characterization was conducted on anatomically-matched skin and fat biopsies as well as serum samples from eleven lipedema and ten BMI-matched healthy patients. Increased systemic levels of vascular endothelial growth factor (VEGF)-C (*P* = 0.02) were identified in the serum of lipedema patients. Surprisingly, despite the increased VEGF-C levels no morphological changes of the lymphatic vessels were observed. Importantly, expression analysis of lymphatic and blood vessel-related genes revealed a marked downregulation of Tie2 (*P* < 0.0001) and FLT4 (VEGFR-3) (*P* = 0.02) consistent with an increased macrophage infiltration (*P* = 0.009), without changes in the expression of other lymphatic markers. Interestingly, a distinct local cytokine milieu, with decreased VEGF-A (*P* = 0.04) and VEGF-D (*P* = 0.02) expression was identified. No apparent lymphatic anomaly underlies lipedema, providing evidence for the different disease nature in comparison to lymphedema. The changes in the lymphatic-related cytokine milieu might be related to a modified vascular permeability developed secondarily to lipedema progression.

## Introduction

Lipedema is a distinct adipose tissue disorder, affecting primarily women^1^. Epidemiological data of large studies are still not available but the prevalence of lipedema is estimated between 7 and 9.7%. Interestingly the prevalence of lipedema in patients referred to lymphatic clinics is increased, estimated as 10–15%^2,3^.

The mechanisms involved in lipedema development are largely unknown and despite the distinct clinical features the disease is commonly misdiagnosed as obesity or lymphedema. Clinically, lipedema is characterized by the bilateral and symmetrical adipose tissue deposition, mostly of the lower extremities, sparing the feet. The Stemmer sign is negative and the edematous appearance is resistant to diet restrictions, elevation of the extremities or lymphatic drainage^4^.

The expansion of the adipose tissue represents the main pathophysiological finding of the disease. In this regard, increased proliferation of adipose stem cells is thought to rapidly increase adipogenesis, resulting in localized hypoxia, adipocyte necrosis and macrophage recruitment^5^. Computer tomography (CT) and magnetic resonance imaging (MRI) of lipedema patients confirm the homogenous lipomatous hypertrophy of subcutaneous tissue^6^.

The lymphatic system plays a pivotal role in the maintenance of tissue homeostasis, immune regulation and absorption of dietary fats^7^. Acting as a circuit of fluid, immune cell and macromolecule transport the lymphatic system critically affects and regulates local tissue environment. Inflammation-associated lymphangiogenesis is common in acute or chronic inflammation and induction of therapeutic lymphangiogenesis can restore acute inflammation^8^. Interestingly, deficient lymphatic architecture or impaired lymphatic function have been reported to cause and result from adipose tissue expansion^9,10^. As such, expansion of the lymphatic vasculature or promotion of its function may offer a mechanism to regulate adipose tissue inflammation and thus influence the aberrant metabolic profile.

Due to the clinical similarities that lipedema and lymphedema patients exhibit, an involvement of the blood or lymphatic vasculature in the onset or development of the disease has been hypothesized. In a study by Siems et al*.* the vascular endothelial growth factor-A (VEGF-A), a potent inducer of angiogenesis and lymphangiogenesis, was found two-fold increased in lipedema patients, resulting in pathological angiogenesis and increased capillary fragility^11^. On the other hand, the presence of a lymphatic vascular component has remained a controversy. Recent work revealed a mild lymphatic phenotype in morbidly obese lipedema patients, whereas obese lipedema patients did not present any morphological differences of the lymphatic vessels^12^. Other imaging studies demonstrated lymphatic microaneurysms in lipedema patients, without macroscopic changes in the lymphatic collecting vessels or lymphatic capillary diameter^13^.

In contrary, the lymphatic phenotype in lymphedema has been thoroughly studied^14^. Lymphedema is defined as the cardinal manifestation of lymphatic vascular dysfunction, occurring in the western world most commonly as result of oncologic surgery. It appears with asymmetrical edema in the affected extremities and good response to the complete decongestive therapy. Over the course of the disease, that takes months or years to develop, the lymphatic vessels become progressively dilated and fibrotic^14,15^. A distinct local cytokine milieu is established, promoting vascular permeability^16^ and a distinct immune cell infiltrate consisting predominantly of CD4 T cells drives the development of lymphedema^17^.

Admittedly, lipedema and lymphedema share similarities in the phenotypic changes occurring in the affected population. What is more, the etiologic role of adiposity in inducing lymphedema as well as the prominent effect of the adipose tissue hypertrophy and/or hyperplasia in both conditions led to the assumption that a lymphatic component is present in lipedema. However the role of the lymphatic system in the development of the disease, remains elusive and scarce histologic and molecular biology data currently exist^18^.

To evaluate the presence of lymphatic manifestation in lipedema, anatomically matched skin and fat probes as well as fasting serum probes were analyzed from lipedema versus gender- and body mass index (BMI)-matched healthy patients undergoing elective plastic surgery operations. A detailed histological and molecular analysis was performed, revealing no lymphatic morphological differences between lipedema and control patients. Interestingly though, a distinct cytokine milieu characterized by systemically increased levels of VEGF-C but decreased expression of VEGF-A and VEGF-D as well as an increased M2 polarized macrophage infiltrate identified might be related to altered permeability of the blood and lymphatic vasculature resulting in microangiopathy.

## Results

### Increased systemic VEGF-C levels in lipedema

**Figure 1:**
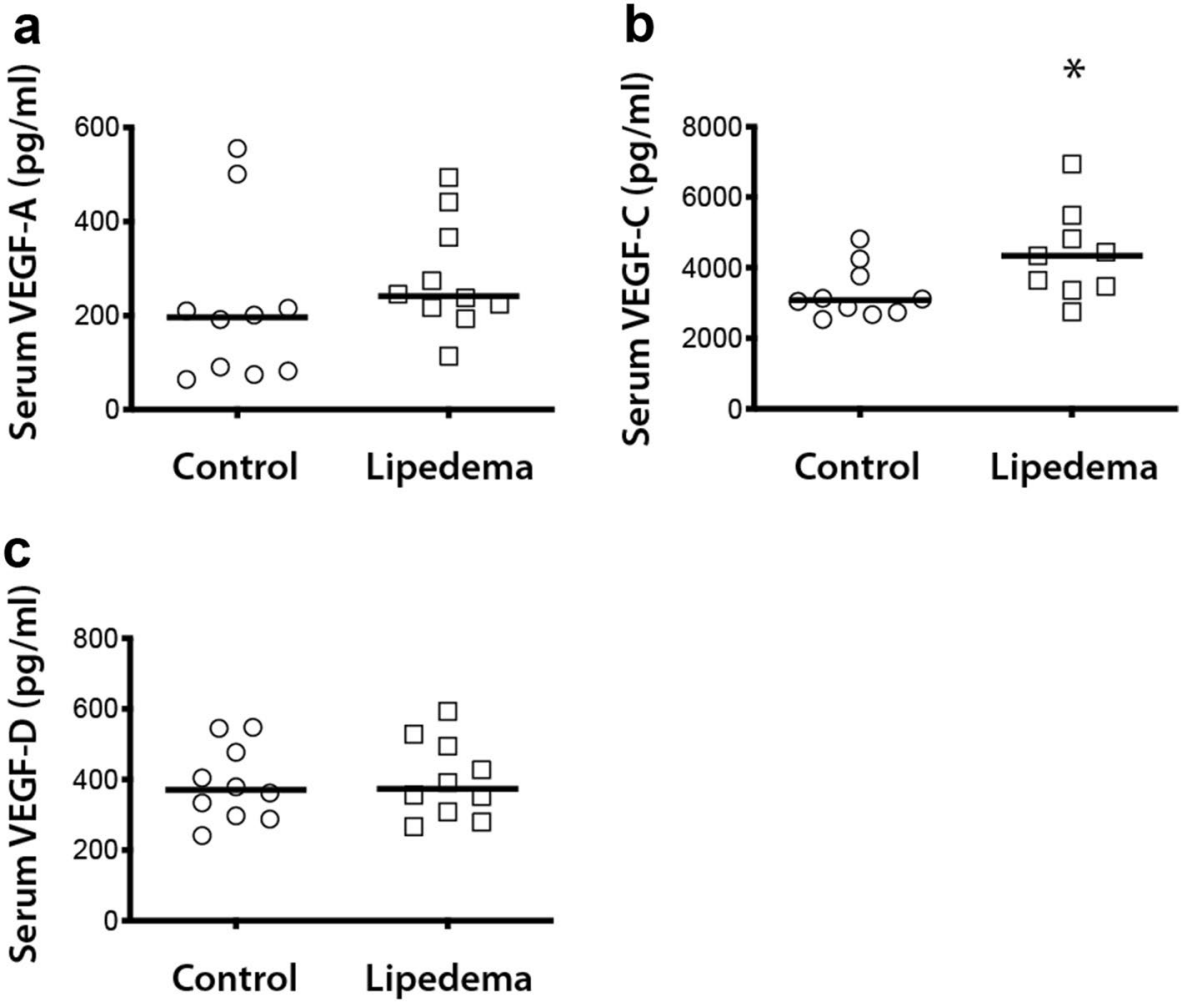
Systemic increase of VEGF-C in lipedema. (**a**–**c**) ELISA of human serum showed increased systemic levels of VEGF-C (C: 3,275 ± 678 pg/ml, L: 4,364 ± 1,204 pg/ml, *P* = 0.02, 1.33-fold increase) but not VEGF-A or VEGF-D in lipedema. N (C): 10 and N (L):10. Asterisks indicate statistical significance in comparison to the control *P < 0.05, (nonparametric unpaired Mann–Whitney *t*-test).

To investigate the potential involvement of lymphatic related cytokines in lipedema, blood serum from lipedema and control patients was collected and evaluated for the three most common cytokines, namely VEGF-A, VEGF-C and VEGF-D. Interestingly, increased levels of VEGF-C were observed in lipedema patients in comparison to the control patients (C: 3,275 ± 678 pg/ml, L: 4,364 ± 1,204 pg/ml, *P* = 0.02, 1.33-fold increase) (Fig. 1b), without any significant differences in the levels of VEGF-A (C: 220 ± 158 pg/ml L: 281 ± 112 pg/ml) and VEGF-D (C: 387 ± 101 pg/ml L: 400 ± 104 pg/ml) (Fig. 1a,c).

### No morphological changes in the lymphatic and blood vasculature in lipedema

**Figure 2:**
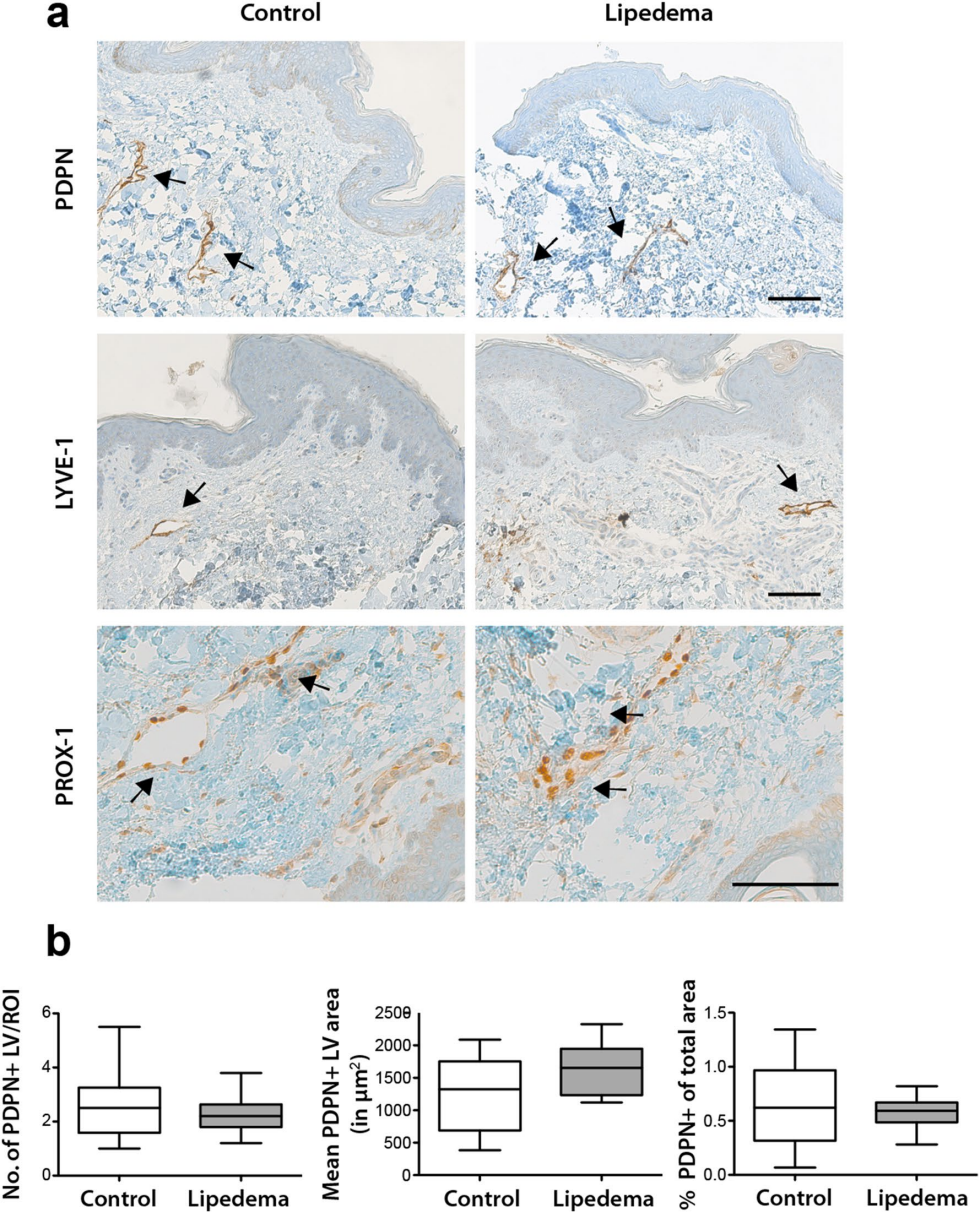
No morphological changes of lymphatic vessels in lipedema. Histological evaluation of the lymphatic vessels on skin sections using three distinct lymphatic markers (PDPN/podoplanin, LYVE-1, PROX-1) revealed no changes in the number, size and percent coverage of lymphatic vessels between control and lipedema patients. N (C): 10 and N (L): 11 (two-tailed Student *t*-test). Scale bar 100 µm.

**Figure 3:**
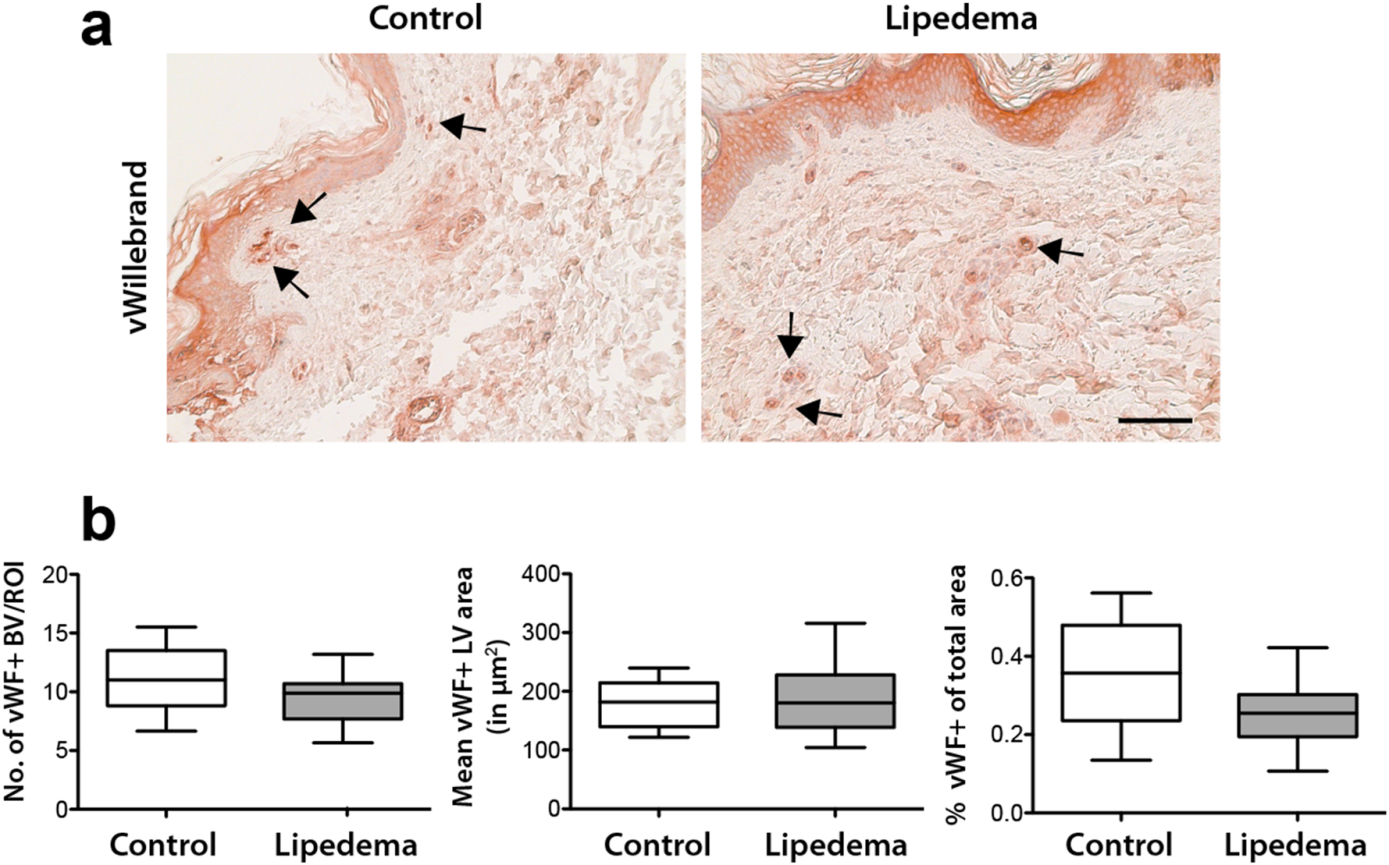
No morphological changes of blood vessels in lipedema. (**a**,**b**) Histological evaluation of the blood vessels on skin sections using an anti-von Willebrand staining revealed no changes in the number, size and percent coverage of blood vessels between control and lipedema patients. N (C): 10 and N (L): 11. (two-tailed Student *t*-test). Scale bar 100 µm.

To evaluate whether the increased systemic levels of VEGF-C result in changes in the lymphatic and blood vasculature, a detailed histological analysis was performed. In this regard, three lymphatic vessel markers (podoplanin/PDPN, LYVE-1 and PROX-1) were used to characterize the size and number of lymphatic vessels in paraffin tissue sections from lipedema and control patients. No changes in the number (C: 2.62 ± 1.24 vessels/field, L: 2.28 ± 0.69 vessels/field), size (C: 1,213 ± 565 µm^2^ L: 1639 ± 380 µm^2^) and percent coverage (C: 0.66 ± 0.4% of field L: 0.57 ± 0.14% of field) of lymphatic vessels in the tissue sections was observed (Fig. 2a,b). To characterize the blood vessels, tissue sections were stained for the von Willebrand factor and the same parameters (number, size and percent coverage of blood vessels) were evaluated, without revealing any differences between the two groups (number of vessels: C: 11.1 ± 2.8 vessels/field L:9.5 ± 2.1 vessels/field, size: C: 178 ± 38 µm^2^ L: 184 ± 60 µm^2^, percent coverage: C: 0.35 ± 0.13% L: 0.25 ± 0.08%) (Fig. 3a,b).

### Distinct gene expression profile in lipedema

**Figure 4:**
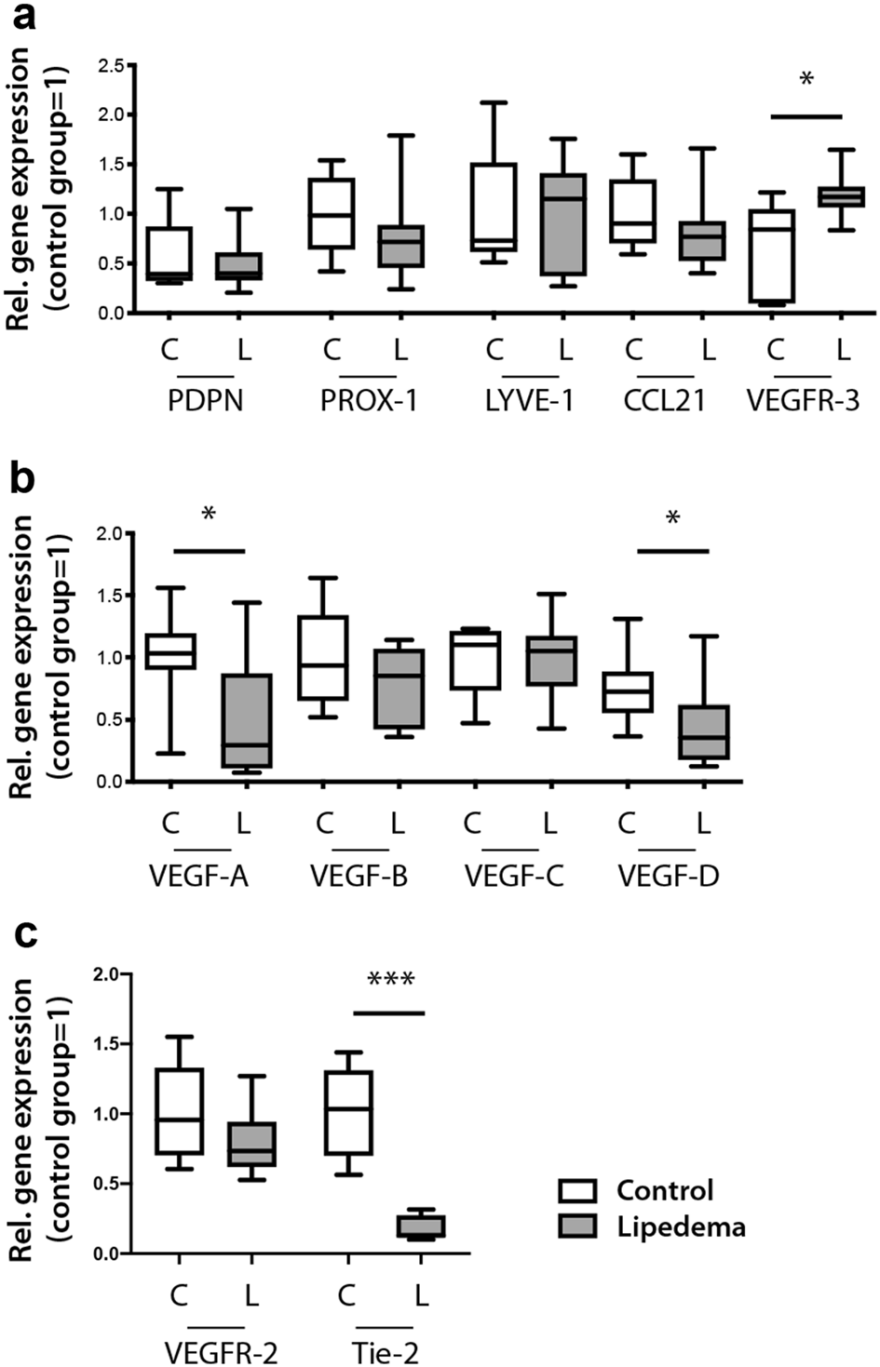
A distinct expression profile of lymphatic-related genes in lipedema. (**a**) Evaluation of the expression profile of the most common lymphatic related genes revealed a 1.9-fold increase in the expression of *VEGFR-3*, while *PDPN*, *PROX-1*, *LYVE-1* and *CCL21* appear unchanged. (**b**) Evaluation of the most common lymphatic-related cytokines revealed a 0.48-fold decrease of the expression of *VEGF-A* and 0.63-fold decrease of the expression of *VEGF-D,* while *VEGF-A and VEGF-B* expression remained unchanged. (**c**) Evaluation of the expression profile of the most common blood vessel-related genes revealed a 5.7-fold decrease in the expression of *Tie2*, while *VEGFR-2* expression remained unchanged. N (C): 5–10 and N(L):10. Asterisks indicate statistical significance in comparison to the control *P < 0.05, ***P < 0.001 (two-tailed Student *t*-test).

To further analyze the potential involvement of the lymphatic and blood vasculature we evaluated the expression profile of the most common lymphatic- and blood vessel-related genes in the adipose tissue using qPCR. No significant changes were observed for most of the common lymphatic markers, namely *PDPN*, *PROX-1*, *LYVE-1*, *CCL21* but a 1.9-fold (*P* = 0.02) increase was observed in the expression of *VEGFR-3* in lipedema in comparison to the control (Fig. 4a). Evaluation of the most common lymphatic-related cytokines revealed a statistically significant 0.48-fold (*P* = 0.04) decrease of the expression of *VEGF-A* and 0.63-fold (*P* = 0.02) decrease of the expression of *VEGF-D* in lipedema in comparison to the control (Fig. 4b). Interestingly, evaluation of common blood vascular markers (VEGFR-2 and Tie2) revealed a significant 5.7-fold (*P* < 0.001) decrease in Tie2 expression in lipedema patients compared to control patients, without any changes in the VEGFR-2 expression (Fig. 4c).

### Increased macrophage infiltration without changes in T cells in lipedema

**Figure 5:**
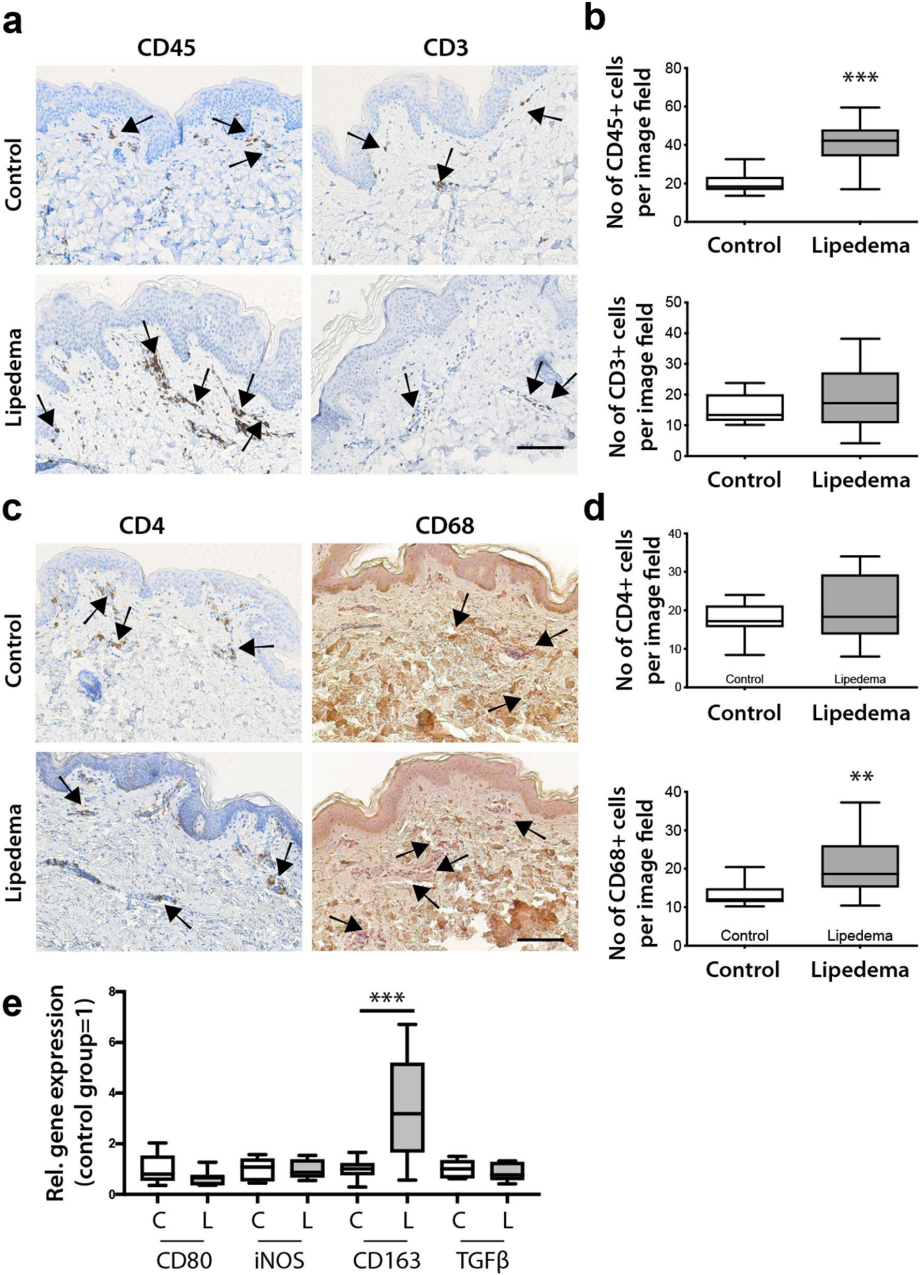
Increased immune cell infiltrate with increased macrophage presence in lipedema. (**a**,**c**) The immune cell infiltrate was evaluated on paraffin embedded tissue sections. The arrows indicate the CD45, CD3, CD4 and CD68 positive cells (**b**,**d**) Quantification of the CD45 + , CD3 + , CD4 + and CD68 + cells reveals an increased infiltration of CD45 + cells. Analysis of the immune cell composition reveals an increase in the macrophage infiltration without affecting the T cell compartment. (**e**) The evaluation of the macrophage polarization using CD80 (M1), iNOS (M1), CD163 (M2) and TGFβ (M2) as markers indicates an M2 phenotype. N(C): 10, N(L): 10 patients. Scale bar: 100 µm. Asterisks indicate statistical significance in comparison to the control **P < 0.01, ***P < 0.001 (two-tailed Student *t*-test).

As *VEGFR-3* is not only expressed on the lymphatic endothelium but on macrophages as well, we lastly sought to evaluate the immune cell composition in lipedema using immunohistochemistry. A two-fold increase in the total immune cell infiltrate (C: 20 ± 4.9 cells/field L: 40.7 ± 10.5 cells/field, *P* < 0.0001) was found in lipedema, using the marker CD45 (Fig. 5a,b). The increased immune infiltrate was not related to any changes in systemic inflammation markers (CRP and number of leukocytes, suppl. Fig. 1a,b).

Analyzing further the immune cell composition and the involvement of lymphoid cells we did not observe any significant change in the T cells compartment (C: 15.5 ± 4.5 cells/field L: 18.5 ± 9.5 cells/field) and CD4 + T cells in particular (C: 17.8 ± 4 cells/field L: 19.5 ± 8.5 cells/field) using the CD3 and CD4 stains respectively (Fig. 5a–d). Finally, evaluation of the macrophage infiltration using the marker CD68 revealed an increased macrophage presence in lipedema (C: 13 ± 2.8 cells/field, L: 21.2 ± 7.8 cells/field, *P* = 0.009) in comparison to the control (Fig. 5c,d). Evaluation of M1 (CD80 and iNOS) and M2 (CD163 and TGFβ) polarization markers using qPCR revealed an increased CD163 expression (3.4-fold increase) in lipedema patients and as such a predominant M2 polarization profile (Fig. 5e).

## Discussion

Lipedema, with an estimated prevalence of 11% in Western countries presents a very common disease that has remained surprisingly understudied. The absence of relevant pre-clinical models constitutes an additional obstacle in understanding the molecular determinants of the disease, hindering the development of therapeutic options. Only recently have histological studies been published, shedding light on the lipedema tissue architecture^12^.

Lipedema is characterized by the aberrant adipose tissue accumulation^19^, particularly of the lower extremities, a situation resembling lymphedema^20^. As such, the assumption that the lymphatic system is involved has been established.

Lipedema as a term defines the state of fat and fluid retention. Imaging studies have proven the accumulation of adipose tissue in the affected extremities^6,21^ and histological studies have revealed prominent adipose tissue hypertrophy^12,19^. The presence of edema, though, has not been verified^22^. Imaging studies and sonography findings in particular could not prove the presence of increased interstitial fluid in lipedema patients^23^. Evaluation of the lymphatic vascular system via MRI lymphangiography in obese lipedema patients revealed microaneurysms without further lymphatic alterations^13^. Therefore, we sought to evaluate the systemic levels of the most common lymphangiogenic cytokines. Interestingly, increased levels of VEGF-C were detected in lipedema patients’ serum, whereas the levels of VEGF-A and VEGF-D remained comparable to the control subjects.

To investigate whether the systemically increased VEGF-C levels are associated with morphological changes in lymphatic and/or blood vasculature, a detailed histological analysis of both vascular networks was performed. No morphological changes in regard to the number, size and percent coverage of the lymphatic and blood vessels were detected using three distinct lymphatic markers (podoplanin/PDPN, LYVE-1 and PROX-1) and one blood vascular marker (vWF). The absence of lymphangiogenesis could be explained by the fact that an approximately 25% increase of VEGF-C in the serum of lipedema patients might not be sufficient to promote lymphangiogenesis. In terms of comparison, the lymphatic vascular remodeling observed in lymphedema has been associated with a 100% increase of the plasma VEGF-C levels^24^. Similar results have been obtained from animal studies, where VEGF-C levels exhibit an approximate 100% increase in the serum of mice with lymphedema versus controls^16^. As such, these results are in line with previous imaging^21,23,25^ studies and indicate that in the absence of morbid adiposity the lymphatic and blood vascular systems are morphologically unaltered in lipedema.

As systemic changes in the cytokine milieu could lead to a modified gene expression pattern, the expression profile of the most common lymphatic and blood vessel genes was investigated. No changes for the majority of the genes was observed, while an increased expression of VEGFR-3 was noticed, combined with prominent Tie2 downregulation as well as reduced expression of VEGF-A and VEGF-D. Tie2 is known to participate in multiple conditions associated with vascular leakage, reacting with a decrease in the activation status and a decline in the abundance of the receptor^26,27^. What is more, VEGF-A and VEGF-D are both known to induce vascular permeability^28,29^ and their reduced expression levels in combination with the significant Tie2 downregulation and the systemically increased VEGF-C levels may present a counterbalancing mechanism to normalize vascular permeability, thus avoiding edema formation.

Given that VEGFR-3 is expressed not only on the lymphatic endothelium but also on the macrophages^30^, an involvement of the macrophages in lipedema was postulated. In an evaluation of the immune cell infiltrate, an increased inflammatory response was noticed in lipedema indeed combined with increased macrophage infiltrate. Interestingly an M2 macrophage polarization phenotype was identified, attributed to the CD163 overexpression. CD163 is hemoglobin/haptoglobin scavenging receptor and its increased expression provides further support to the hypothesis of a compromised vascular permeability with extravasation of red blood cells, necessitating an increased presence of macrophages with scavenging functions. These results are in line with previous reports and underline the potential role of macrophages in lipedema^12^. In contrary, no change in the T cell component was noticed in lipedema, a noticeable difference in comparison to lymphedema, where the T cell component was shown to play a pivotal role in the onset and development of the disease^17^.

In summary, this is the first study, to the best of our knowledge that comprehensively shows that no morphological alterations of the lymphatic and blood vascular system underlie lipedema development. The histological results as such further confirm previous clinical observations that no true edema is present in lipedema. The increased systemic levels of VEGF-C might be related to the increased macrophage infiltrate, whereas the decreased expression of Tie2, VEGF-A and VEGF-D could be a counterbalancing mechanism to regulate vascular permeability. The histological results coupled with the gene expression data indicate that the distinct cytokine milieu might be secondary to lipedema development and largely related to the distinct macrophage infiltrate. Despite the small number of patients, this study presents a very homogenous patient group and is the first study to characterize the lymphatic involvement using systemic markers as well as histologic and molecular biology methods.

The study also provides first evidence that no lymphatic phenotype underlies lipedema development and as such treatment using lymphatic drainage/complete decongestive therapy or lymphatic correction operations may not present feasible options for lipedema treatment. Further work should be focused on elucidating the role of macrophages in lipedema development and particularly on their role in adipose tissue metabolism.

## Materials and methods

### Patients

The protocols of the current study were approved prior to patient recruitment by the Ethical Committee of the University Hospital Goettingen, State of Lower Saxony, Germany (Nr. 23-11-17) and the study has been conducted according to the principles of the Declaration of Helsinki. All patients were informed in detail prior to the surgical procedures in oral and written form and provided their written informed consent. The samples were obtained from lipedema and BMI- as well as age-matched control female patients. Lipedema was diagnosed based on the criteria of Wold et al.^1^, namely (1) presence predominantly in women, (2) bilateral increase of the adipose tissue of the lower extremities sparing the feet, (3) negative Stemmer’s sign, (4) pain, tenderness and tendency to bruise, (5) persistence to weigh loss or extremity elevation. All patients fulfilled these criteria to be operated and included in the analysis. Skin and fat tissue derived from the proximal part of the lower extremities of the thigh, as anatomically matched biopsies.

The patient characteristics are presented in suppl. Table 1.

### Tissue collection and histology

Skin and adipose tissue specimens were collected during the operating procedure and were directly fixed for 4 h in 4% PFA/PBS at 4 °C. Subsequently the samples were embedded in paraffin. 5 µm thick tissue sections were used for the histological analysis.

Images were obtained using a Leica Leitz DM RXE microscope equipped with a Leica DFC490 camera and up to 5 images per tissue were acquired using a PL Fluotar 20×/0.5 NA or PL Fluotar 40×/0.7 NA objective. Morphometric analysis of the lymphatic vessels (lymphatic vascular area coverage and number of lymphatic vessels per field) and immune cells (number of immune cells per field) was performed using ImageJ software (National Institutes of Health, Bethesda, MD) by a blinded examiner.

### Immunohistochemistry

5 µm thick paraffin sections were stained at the Department of Pathology of the University Hospital Zurich according to standardized protocols. First paraffin embedded sections were deparaffinized and rehydrated. Lymphatic vessel stainings were performed using an automated Ventana BenchMark Ultra, mouse anti-human D2-40 (podoplanin) (Agilent Dako, Santa Clara, CA, USA, M3619; clone D2-40, 1:50), polyclonal goat anti-human anti-LYVE-1 (R&D, Minneapolis, MN, USA, AF2089, 1:100) polyclonal goat anti-human PROX-1 (R&D, Minneapolis, MN, USA, AF2727, 1:50) with antigen retrieval with Tris–Borate buffer for 32 min.

The immune cell evaluation was performed at the Department of Pathology of the University Hospital Goettingen. Briefly, CD45, CD3 and CD4 stains were performed using an automated Ventana BenchMark Ultra and the following antibodies: CD45, CD3, CD4 with antigen retrieval with Tris–Borate buffer for 30 min.

For the CD68 and von Willebrand factor (vWF) stain, tissue sections were first deparaffinized and rehydrated. Antigen retrieval was performed with Proteinase K (Agilent Dako, Santa Clara, CA, USA, S3020) and endogenous peroxidase activity was blocked using Bloxall (Vector SP-6000). After blocking (Vectastain mouse-HRP-kit, Vector PK-6102 plus 1.5% horse serum) for 1 h at room temperature the sections were incubated with mouse anti-human CD68 antibody (Agilent Dako, Santa Clara, CA, USA, M0876, clone PG-M1, 1:100) or rabbit anti-human vWF antibody (Agilent Dako, Santa Clara, CA, USA, polyclonal, 1:100) at 4 °C on a shaker overnight. The detection was performed using the Vectastain Kit with AEC substrate, according to the manufacturer’s instructions.

### RNA extraction and qPCRs

RNA was isolated from fat tissue using the RNeasy Lipid Tissue Mini Kit Qiagen (Cat no. 74804). cDNA was transcribed from ca. 500 ng RNA template with the cDNA RT2 First Strand Kit from Qiagen (Hilden, Germany, Cat no. 330404). The primers used to evaluate gene expression are provided in a separate table (suppl. Table 2). FastStart SYBR green master mix (Roche) was used for the PCR reactions. A CFX 96 C100 Thermal Cycler was used; B2M served as housekeeping gene and fold changes of gene expression were calculated using the ΔΔ_CT_ methody^31^.

### Serum isolation and ELISAs

For the cytokine evaluation blood was collected in a S-Monovette (Sarstedt, Nuernbrecht, Germany) preoperatively and upon 8–10 h of starvation. Blood was left to coagulate for 30 min at RT and then was centrifuged for 10 min at 1000*g*. Serum was aliquoted and stored at − 150 °C until usage.

Cytokine quantification in the human serum samples was performed according to the ELISA kit manufacturer’s directions (human VEGF; Cat. DVE00, human VEGF-C; Cat. DVEC00, human VEGF-D, DVED00, all from R&D, Minneapolis, MN, USA).

### Statistical analysis

All data represent mean ± SD, depicted in whisker plots exhibiting the 5–95 percentiles. Means of two groups were compared as follows: D'Agostino-Pearson omnibus normality test was performed to evaluate normal distribution of the data. Outliers have been excluded from the analysis. A nonparametric unpaired Mann–Whitney *t*-test was performed for non-Gaussian distribution, whereas a two-tailed Student *t*-test was performed for Gaussian-distribution. Sample sizes and statistical analyses are indicated in the figure legends, unless otherwise mentioned. Statistical analyses were performed using GraphPad Prism V8.0 (GraphPad Software, San Diego, CA, USA). *P* < 0.05 was accepted as statistically significant.

## Supplementary information

Supplementary Figure 1

Supplementary file2
