# Supplementary figures and images for: Increased levels of VEGF-C and macrophage infiltration in lipedema patients without changes in lymphatic vascular morphology
Source: Sci Rep. 2020 Jul 2;10:10947. doi: 10.1038/s41598-020-67987-3 (PMC7331572; doi:10.1038/s41598-020-67987-3)

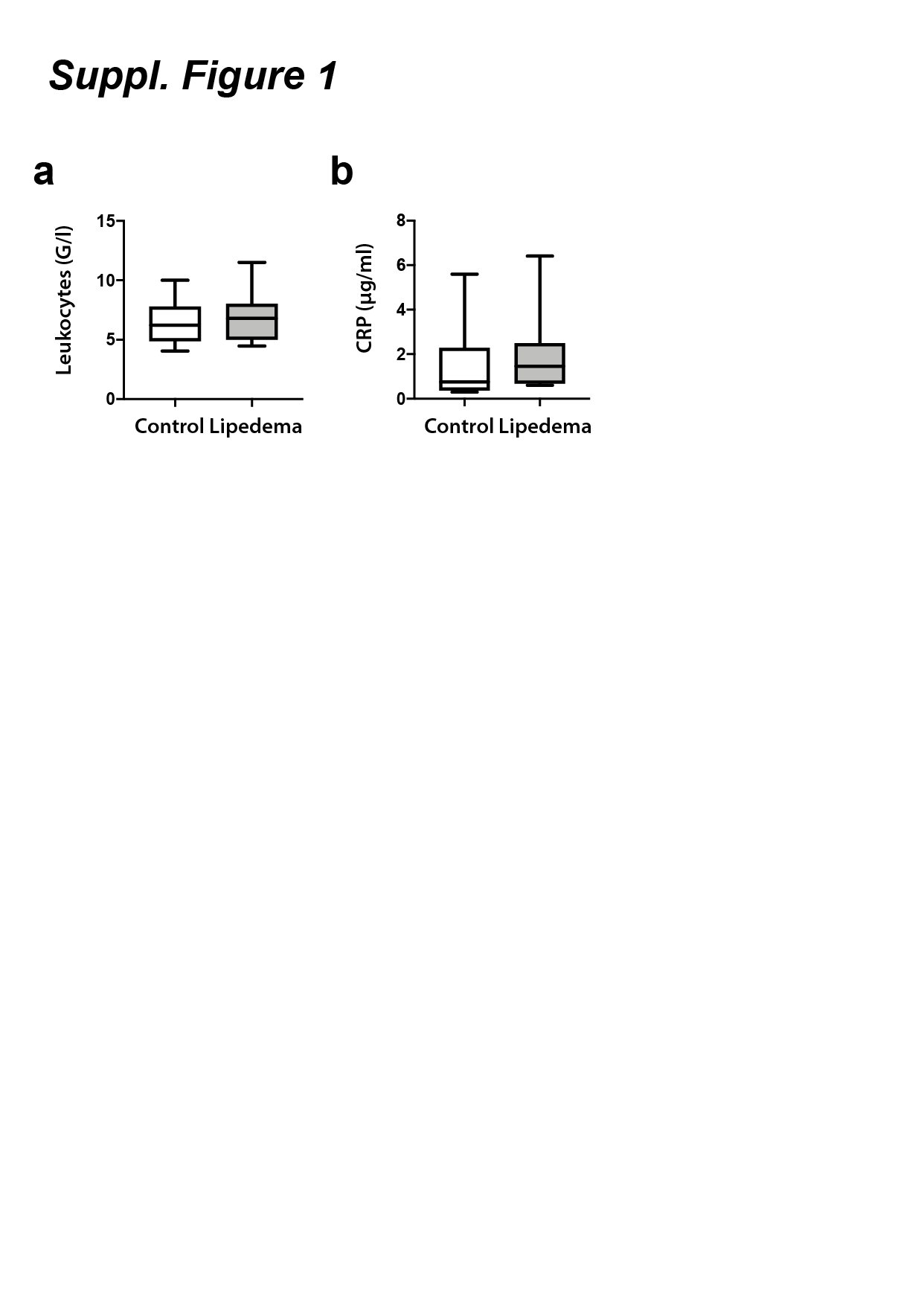

Supplement: Supplementary file 1 — Supplementary Figure 1 [file 41598_2020_67987_MOESM1_ESM.png]
