# Supplementary material for: Increased levels of VEGF-C and macrophage infiltration in lipedema patients without changes in lymphatic vascular morphology
Source: Sci Rep. 2020 Jul 2;10:10947. doi: 10.1038/s41598-020-67987-3 (PMC7331572; doi:10.1038/s41598-020-67987-3)
Supplement: Supplementary file 2 — Supplementary file2 [file 41598_2020_67987_MOESM2_ESM.docx]

**SUPPLEMENTAL MATERIAL**

**Increased levels of VEGF-C and macrophage infiltration in lipedema patients without changes in lymphatic vascular morphology**

Gunther Felmerer^1^*, Aikaterini Stylianaki^1,2^*, Maija Hollmén^3^, Philipp Ströbel^4^, Adam Stepniewski^1^, Anna Wang^5^, Florian S. Frueh^5^, Bong-Sung Kim^5^, Pietro Giovanoli^5^, Nicole Lindenblatt^5^, Epameinondas Gousopoulos^5^

^1^Division of Plastic Surgery, Department of Trauma Surgery, Orthopaedics and Plastic Surgery, University Medical Center Göttingen, Georg-August-University, Göttingen, Germany

^2^ Department for Plastic, Aesthetic and Hand Surgery, General Hospital Braunschweig, Brunswich, Germany

^3^ MediCity Research Laboratory, University of Turku, Turku, Finland

^4^ Institute of Pathology, University Medical Center Göttingen, Georg-August-University, Göttingen, Germany

^5^ Department of Plastic Surgery and Hand Surgery, University Hospital Zurich, Zurich, Switzerland

*These authors contributed equally

Corresponding author:

Epameinondas Gousopoulos, MD, PhD

Department of Plastic Surgery and Hand Surgery

University Hospital Zurich,

Rämistrasse 100

8091, Zurich, Switzerland

Tel. +41 43 25 38 371

Email: [epameinondas.gousopoulos@usz.ch](mailto:epameinondas.gousopoulos@usz.ch)

**Suppl. Table 1**

**Patient characteristics**

| Number of cases | 21 |
| --- | --- |
| Lipedema patients | 11 |
| Control patients | 10 |
| Gender |  |
| Female | 21 |
| Male | 0 |
| Mean age (in years) |  |
| Lipedema patients | 40.8 ±7.2 |
| Control patients | 47.9 ±9.7 |
| Mean weight (in kg) |  |
| Lipedema patients | 77.1 ±7.1 |
| Control patients | 78.4 ±14.3 |
| Mean BMI (in kg/m^2^) |  |
| Lipedema patients | 27.3 ±2.01 |
| Control patients | 27.8 ±4.28 |
| Lipedema Staging |  |
| Stage I | 0 |
| Stage II | 4 |
| Stage III | 7 |
| Stage IV | 0 |

**Suppl. Table 2**

**List of primers**

| VEGF-for | CTACCTCCACCATGCCAAGT |
| --- | --- |
| VEGF-rev | GCAGTAGCTGCGCTGATAGA |
| VEGFB-for | GAGATGTCCCTGGAAGAACACA |
| VEGFB-rev | GAGTGGGATGGGTGATGTCAG |
| VEGF-C -for | CACCACCAAACATGCAGCTG |
| VEGF-C-rev | TGAAAATCCTGGCTCACAAGC |
| VEGFD-for | ATGGACCAGTGAAGCGATCAT |
| VEGFD-rev | GTTCCTCCAAACTAGAAGCAGC |
| PROX1-for | ACAAAAATGGTGGCACGGA |
| PROX1-rev | CCTGATGTACTTCGGAGCCTG |
| LYVE-1-for | AGCTATGGCTGGGTTGGAGA |
| LYVE-1-rev | CCCCATTTTTCCCACACTTG |
| PODOPLANIN (PDPN)-for | AGGCGGCGTTGCCAT |
| PODOPLANIN (PDPN)-rev | GTCTTCGCTGGTTCCTGGAG |
| FLT4 (VEGFR-3)-for | TCTGCTACAGCTTCCAGGTGG |
| FLT4 (VEGFR-3)-rev | GCAGCCAGGTCTCTGTGGAT |
| CCL21-for | GGTTCTGGCCTTTGGCATC |
| CCL21-rev | AGGCAACAGTCCTGAGCCC |
| VEGFR2-for | GGC CCA ATA ATC AGA GTG GCA |
| VEGFR2-rev | TGT CAT TTC CGA TCA CTT TTG GA |
| Tie2-for | TGTTCCTGTGCCACAGGCTG |
| Tie2-rev | CACTGTCCCATCCGGCTTCA |
| CD80-for | CTGCCTGACCTACTGCTTTG |
| CD80-rev | GGCGTACACTTTCCCTTCTC |
| iNOS-for | ATGCCCGATGGCACCATCAGA |
| iNOS-rev | TCTCCAGGCCCATCCTCCTGC |
| CD163-for | ACATAGATCATGCATCTGTCATTTG |
| CD163-rev | ATTCTCCTTGGAATCTCACTTCTA |
| TGFβ-for | CCCAGCATCTGCAAAGCTC |
| TGFβ-rev | GTCAATGTACAGCTGCCGCA |
